# Advances in clinical applications of microneedle

**DOI:** 10.3389/fphar.2025.1607210

**Published:** 2025-06-26

**Authors:** Kequan Chen, Xinyu Sun, Yangkaiyuan Liu, Shiyu Li, Dianhuai Meng

**Affiliations:** ^1^ The Second Clinical Medical School of Nanjing Medical University, Nanjing, China; ^2^ School of Biological Science & Medical Engineering, Southeast University, Nanjing, China; ^3^ The First Clinical Medical School of Nanjing Medical University, Nanjing, China; ^4^ Rehabilitation Center, The First Affiliated Hospital with Nanjing Medical University, Nanjing, China

**Keywords:** microneedles, transdermal drug delivery, biosignal detection, cancer treatment, personalized medicine

## Abstract

Microneedle (MN) technology, characterized by its micron-scale structure, can effectively break through the skin barrier, enhance the efficiency of transdermal drug delivery and achieve precise biosignal detection. Research indicates that MNs demonstrate superior safety and efficacy in clinical applications, significantly improving drug delivery efficiency, enhancing patient compliance and reducing side effects. In the field of biosensing, the combination of MN arrays and biosensors enables highly sensitive real-time monitoring of biomarkers. In cancer treatment, MNs exhibit potential for targeted drug delivery, gene therapy, and immunostimulation. Moreover, MNs present broad prospects in wound healing, scar repair, anti-aging and skin disease treatment. This review aims to systematically summarize recent advances in MNs applications across transdermal drug delivery, biosensing, cancer therapy, and skin disease repair through recent high-quality studies, and to explore future development prospects.

## 1 Introduction

**FIGURE 1:**
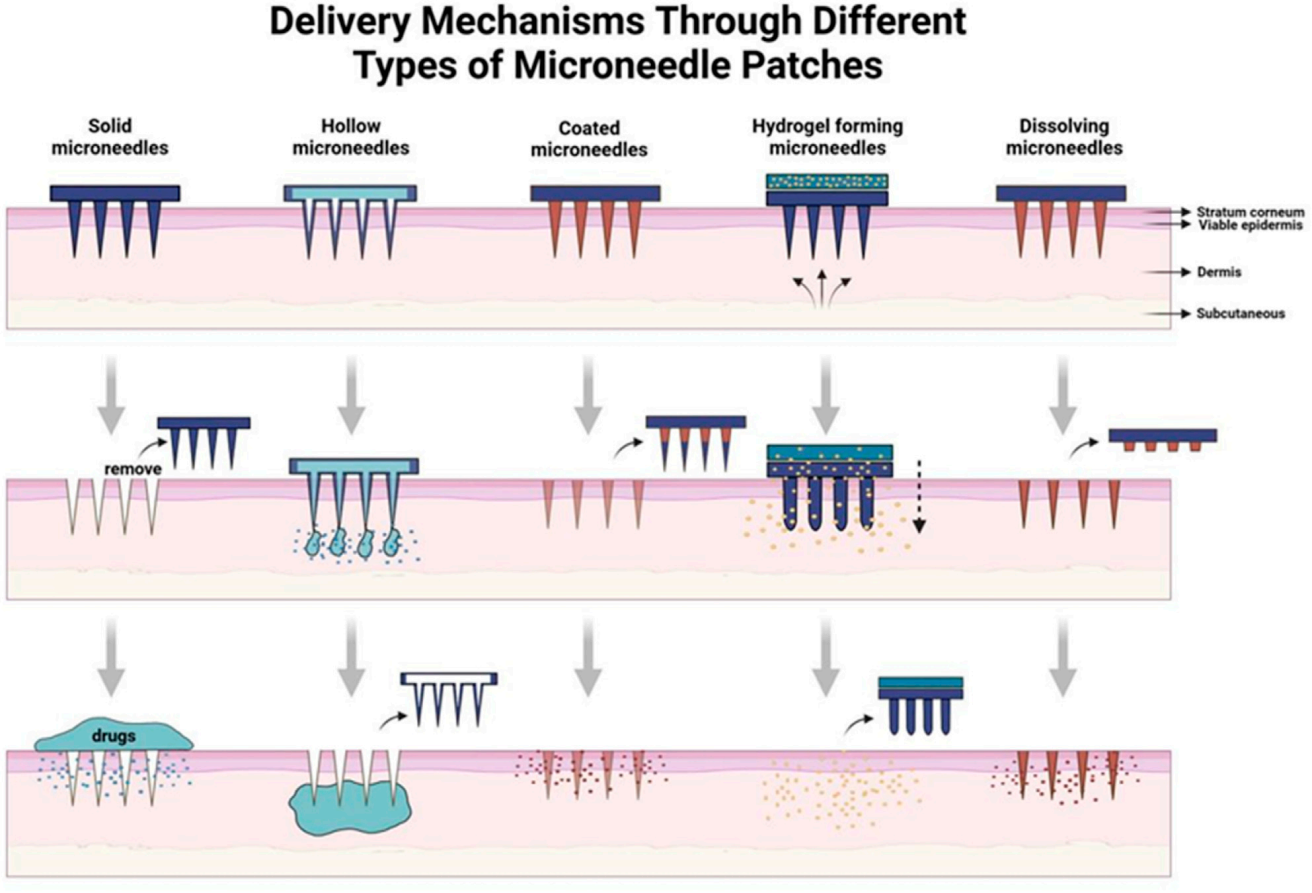
Brief introduction to delivery mechanisms through different types of MN patches.

Drug delivery technologies have facilitated the development of numerous therapeutics, and various formulations enhance patient outcomes by improving targeted delivery, minimizing off-target accumulation, and promoting compliance (Zhuang et al., 2024). An effective drug delivery technology not only fulfills its therapeutic objectives but also enhances the overall patient experience. Such a need has given rise to MNs, an innovative platform that goes beyond the traditional mode of drug delivery methods (Figure 1).

MNs were developed by Henry et al., in 1998 using microfabrication techniques. They consist of an array of fine needles (100–1,000 μm in length) attached to a base (Larrañeta et al., 2016). Subsequently, Henry et al. applied MNs for transdermal delivery of calcineurin Currently, MNs have been extensively reported for transdermal delivery of small molecule drugs, nucleic acids, peptides, proteins and other substances (Weng et al., 2023). With the development of science and technology, MNs, as a novel minimally invasive transdermal technology, have gradually gained attention in the medical field. In 2020, microneedling was ranked as the first of the top ten emerging technologies that were expected to transform the world by Scientific American, an authoritative science magazine (Fu et al., 2024).

MNs can break through the skin’s stratum corneum - the main barrier to transdermal drug delivery - in a minimally invasive way. They consist of small, sharp micrometer-sized arrays, which leave microchannels in the skin after penetrating the skin, and can improve the permeability of drug molecules through the skin (Li et al., 2025). MNs offer several advantages, including minimal invasiveness and pain, rapid onset of action, safety, independence from molecular size or polarity, and high patient compliance (Shin et al., 2022). First pass elimination and gastrointestinal reactions can also be avoided compared to oral administration (Liu et al., 2022). In addition to large-scale transdermal drug delivery, MNs have also been widely used in vaccination, diagnostic testing, medical cosmetology and other biomedical fields in recent years (Zhuang et al., 2024). MNs, initially developed as a replacement for traditional syringes, have gradually evolved toward theranostic integrations. The range of MN-based technologies continues to expand, and its future development remains highly anticipated.

Currently, numerous researchers have conducted comprehensive reviews on various applications of MNs, including specialized analyses of bioelectronic sensors (Vora et al., 2023) or molecular biomarker detection (Li et al., 2024). Compared to these existing studies, our work provides a concise overview of MNs clinical applications while incorporating more recent research advancements. We systematically analyze the strengths and limitations of MNs, aiming to provide methodological insights that may inform future research and innovation in this domain.

## 2 Transdermal drug delivery

Transdermal drug delivery systems mediated by MN patches have a higher acceptability and safety compared to conventional drug delivery systems. While traditional transdermal drug delivery systems can avoid gastrointestinal and first-pass reactions compared to oral drug delivery, the skin barrier can limit the efficiency of drug delivery. MN patches, on the other hand, can painlessly penetrate the skin barrier without causing pain, increasing the efficiency of drug delivery and improving patient compliance (Larrañeta et al., 2016). Currently, key challenges include poor control over delivery depth, inadequate regulation of immune effects, and insufficient responsiveness at target sites. If there is a breakthrough in delivery depth control, immune balance regulation and industrialization, the modular design of MNs provides a new paradigm for the transformation of traditional drug delivery and personalized tumor immunotherapy.

### 2.1 Vaccines

#### 2.1.1 Cancer vaccines

Weng et al. were the first to employ a peptide vaccine (TMV-PEP3) conjugated with tobacco mosaic virus (TMV) for immunotherapy of tumors such as triple-negative breast cancer delivered via dissolving MNs to effectively induce specific antibodies and cytotoxic responses. This study used MNs in combination with a nanovaccine targeting DCs for the treatment of triple-negative breast cancer, which successfully avoided conventional targets, fully utilized dendritic cells as an abundant targeting resource in the skin, and effectively blocked the low-target rate-limiting phase of tumor immunity (Weng et al., 2023). In addition, researchers used Prussian blue nanoparticles to induce immunogenic cell death of tumor cells followed by delivery to subcutaneous T cells via porous gelatin MNs to activate anti-lymphoma immune responses. The key advantage of this achievement lies in the controllable manufacturing of cancer cells (Fu et al., 2024). However, uniform dispersion of nanoparticles in the MN matrix still remains a challenge.

Recently, in order to promote dendritic cell activation, significantly enhance immune response to tumor antigens (e.g., ovalbumin), and delay tumor growth, Li et al. (2025) were inspired by ice-pop to fabricate photothermal ultra-swelling MN (PUSMN), which triggered localized photothermal effects through near infrared, which was just enough to achieve the effect. Given its ease of use, efficiency and safety, this biocompatible PUSMN patch could greatly improve cancer vaccination. However, the depth of thermal penetration of photothermal therapy needed to be validated to match the tumor site.

#### 2.1.2 Vaccines against viral diseases

Starting with the common influenza virus, researchers had targeted the persistent mutations of the H3N2 strain. Considering the time-sensitivity of influenza protection, they had combined high-performance liquid chromatography (HPLC) with MN-based injection, effectively providing protection against multiple antigen variants (Shin et al., 2022). Furthermore, researchers had demonstrated in a mouse model that dissolving MN H7N9 vaccines had a long *in vivo* retention of virus antigen compared to traditional intramuscular injections and may serve as an effective route of immunization (Liu et al., 2022).

**FIGURE 2:**
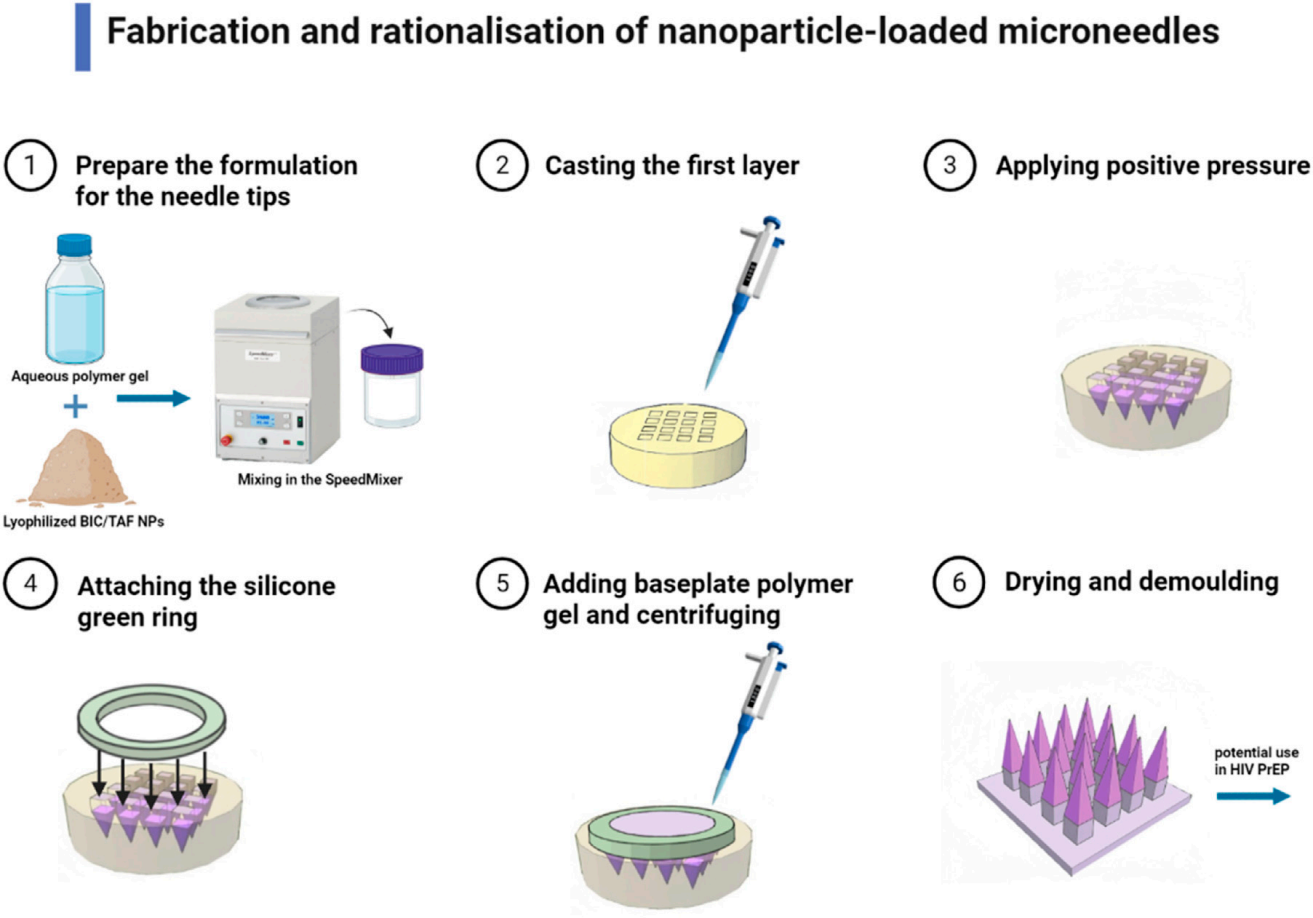
Fabrication and rationalization of nanoparticle-loaded MNs (Dissolving MNs containing either BIC or TAF SDNs were fabricated in a two-step process. Needle tips were formulated by mixing lyophilized BIC/TAF SDN with an aqueous polymer blend in a 1:1 weight ratio. The aqueous mixture was then homogenized with a Speedmixer, and poured onto a silicone MNs mould to cast the first layer of the MN array. The second layer consisted of prefabricated silicone green ring inserts fixed to the mold surface with aqueous substrate gel. This dissolving MN can be used in HIV PrEP for long-lasting plasma concentration maintenance) (Zhang C. et al., 2024).

In addition, fluoropolymer-modified nanovaccines (FiR MNVs) for rabies were delivered via MN, significantly enhancing neutralizing antibody levels and providing 6 weeks of complete protection (Deng et al., 2023). Dissolving MN patch system for AIDS (HIV) delivered Bictegravir and Tenofovir prodrugs for long-lasting plasma concentration maintenance as an alternative to oral pre-exposure prophylaxis (Figure 2) (Zhang C. et al., 2024). Qβ Virus-like Particle (VLP) MN vaccine for HPV Virus is room-temperature stable, induced neutralizing antibodies through a dose-sparing effect, and supported self-vaccination (Ray et al., 2022). Dry-coated chimeric dengue virus vaccine for dengue high-density microarray patch (HD-MAP) retained 100% efficacy after 6 months of 4°C storage (Choo et al., 2021). Polyphosphazene-based MNs for Ebola virus loaded on glycoprotein antigens to induce long-lasting antibodies that completely protect mice against lethal doses of virus (Romanyuk et al., 2023). These different types in MNs had accomplished the protection of their autoimmune effects, and it could be said that MNs had not only achieved protection in transportation but also surpassed immunity after vaccination.

#### 2.1.3 Vaccines against bacterial diseases

*Neisseria gonorrhoeae*, the bacterium responsible for gonorrhea infections, had produced a significant number of gonorrhea patients globally and had gradually developed resistance to anti-microbial drugs over the years. Bagwe et al. evaluated vaccine efficacy by delivering inactivated compound gonococcal microparticles adjuvant (Alum/AddaVax) via MN to female mice, inducing the production of mucosal IgA and serum bactericidal antibodies. The mice showed enhanced expression of CD4 and CD8 cells in the spleen and lymph nodes, demonstrating a cellular immune response and accelerated bacterial clearance. This result broadens the understanding of the immune pathway of gonorrhea and demonstrated strong immunogenicity (Bagwe et al., 2023).

The investigators developed a method for MNs based on 30% w/w poly (methyl vinyl ether-alt-maleic anhydride) water mixtures. Both empty and antigen-loaded MNs were prepared using the outer membrane vesicles of *Shigella* flexneri as an antigenic model. In vivo immunization and conservation studies demonstrated that trans-auricular intradermal immunization of mice with MNs containing 200 µg of the antigenic complex triggered the production of specific systemic IgG and mucosal IgA, which protected the mice against experimental *Shigella* flexneri infections after 4 weeks of immunization. This study demonstrated for the first time the potential of dissolving MNs loaded with outer membrane vesicles for intradermal vaccination against enteric pathogens such as *Shigella* (Pastor et al., 2019). It should be noted that the antigen-antibody complex required for this study has a large drug loading capacity, and in the future, it would be necessary to reduce the drug loading capacity to achieve the corresponding effect. At the same time, the rate of degradation of the hydrogel matrix needed to be matched with the antigen to optimize drug activity and release profiles. In tuberculosis prevention, scientists developed dissolving MNs for the delivery of Ag85B DNA vaccine. High-dose MN vaccination elicited a better antibody response than traditional intramuscular injection, and also led to higher levels of IFN-γ and TNF-α produced by splenic lymphocytes. Animal experiments showed significant reductions in viable counts and prolonged survival in the lungs and spleens of the high-dose MN-inoculated group of mice. This suggested that MN inoculation might provide more effective protection than intramuscular injection. However, to enhance the immunization effect of MN-delivered Ag85B DNA vaccine, improvements such as increasing the MN loading and adding suitable adjuvants deserved further investigation (Yan et al., 2018).

### 2.2 Contraceptive

**FIGURE 3:**
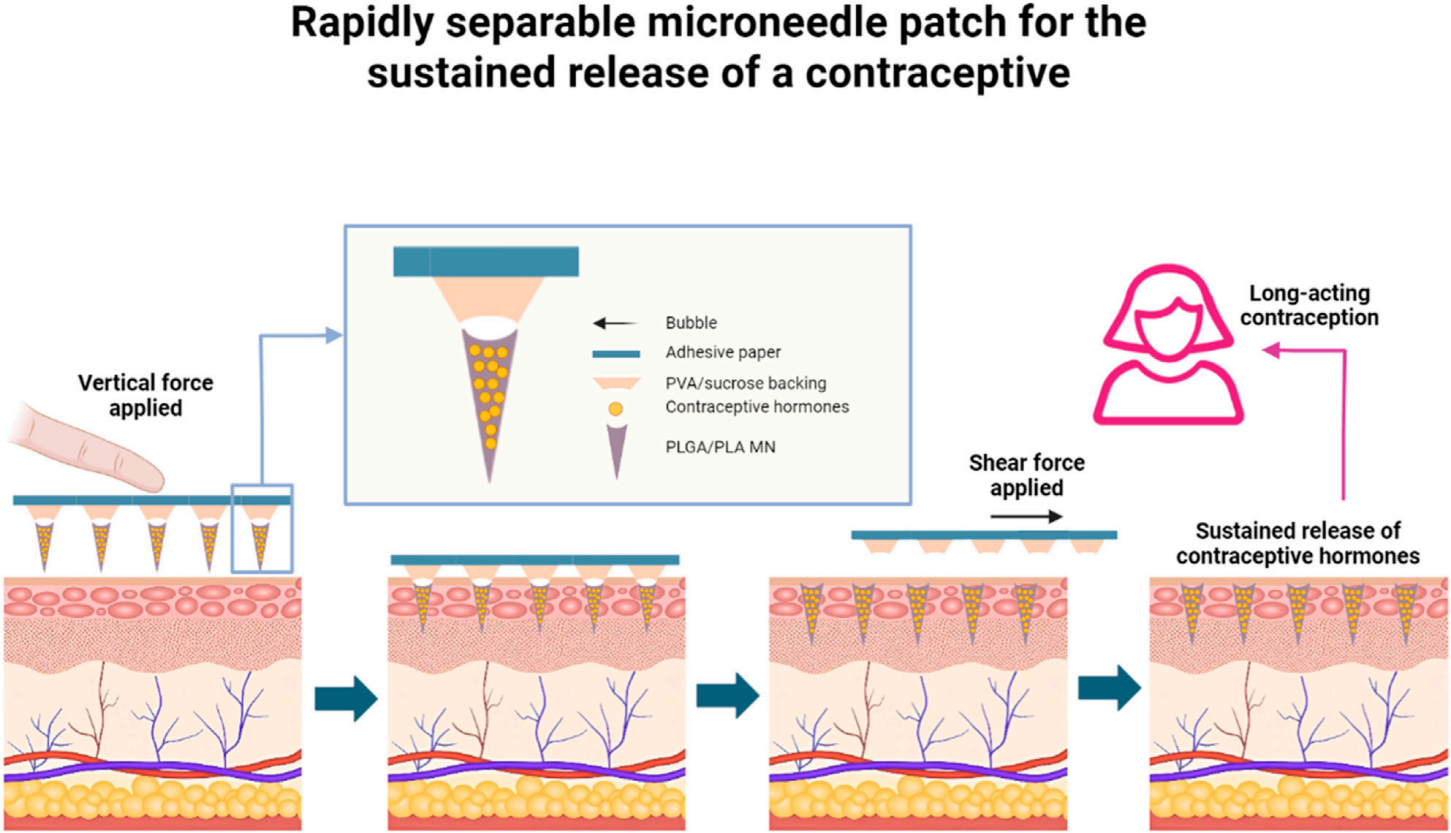
Rapidly separable MN patch for the sustained release of a contraceptive (A MN patch with rapidly separable biodegradable polylactic acid and polylactic-co-glycolic acid needles can continuously release levonorgestrel. Bubble structures between each MN and the patch backing allow the MNs to efficiently penetrate skin under compression, and to snap off under shear within 5 s after patch administration, leaving the MNs inside the skin, which allows for the slow and safe release of levonorgestrel in the body over weeks or even months, providing long-lasting contraceptive efficacy) (adapted with permission from Li et al., 2019. Copyright © 2019, The Author(s), under exclusive licence to Springer Nature Limited).

Although contraceptive methods had improved considerably, there were about 121 million unintended pregnancies in women globally each year from 2015 to 2019 (Bearak et al., 2020). Unintended pregnancies impose a huge economic and emotional burden on women and society, mainly because existing contraceptive methods could not satisfy the needs of different women in different situations, Prausnitz and his co-workers (Figure 3) (Li et al., 2019) demonstrated a MN patch made of safe biodegradable material, which can be briefly and painlessly applied to the skin by the patient, and then the MN could be broken and embedded in the skin to release the contraceptive pill continuously for a period of time. The MN not only solved the problem of patient accessibility, low maneuverability and limited safety of some contraceptives, but also synchronizes material degradation with the contraceptive cycle, avoiding drug residues, and was able to satisfy the patients’ needs for long-term contraception. The only caveat is that the safety issue for MN-carrying contraceptives needed to be properly addressed when they are put into use.

### 2.3 Anesthesia

In modern society, pain management was a rather challenging issue, and it was crucial to ensure a fast, effective approach while providing patients with a relatively comfortable experience. Babakurd et al. (2024) used MN patches dissolved with anesthetic EMLA cream for preoperative management of children requiring palatal anesthesia. They then conducted a randomized controlled clinical trial to evaluate the effectiveness, which showed that the anesthetic MN patches significantly reduced the pain of anesthesia in children and had the potential to improve compliance compared to conventional palatal injections. Liu X. et al. (2024) developed a mosquito proboscis-inspired cambered MN patch for anesthesia of the ocular region. The patch creates MN apertures in the cornea that healed completely within 24 h, and the mosquito proboscis-inspired structure made the patient barely feel any significant pain, potentially offering a minimally invasive yet effective alternative delivery system.

Recently, Mao et al. (2024) used a dissolvable hollow MN casing allowing the anesthetic, such as lidocaine hydrochloride, to be quickly released into the skin upon insertion, achieving rapid analgesic effects. This approach had already been validated in rats. Similarly, Wang’s team integrated an iontophoresis-driven device, controlled by a smartphone, into conventional dissolvable MNs, enabling patients to accomplish self-control of regional pain in the body. This strategy of combining dissolving, infiltrating, and controllable iontophoresis therapy had largely contributed to the precision and controllability of MN drug delivery (Wang et al., 2025).

### 2.4 Specialized types of drug administration

Zhong et al. (2024) used a drug-loaded MN array to deliver riboflavin to the posterior sclera. The μ-LEDs embedded in the patch emitted blue light, inducing collagen cross-linking, which strengthened the sclera and maintained its thickness, thereby slowing the recurrence of myopia caused by scleral thinning and stretching. In experiments on pigs and New Zealand rabbits, no structural or degenerative changes were observed, demonstrating short-term safety and laying the foundation for potential myopia treatment in humans. However, this study did not assess the potential phototoxicity of blue light on the retina after long-term exposure and did not explore the possible effects of changes in scleral elasticity after cross-linking on the regulation of ocular physiological functions. In addition, this technique required a combination of MN implantation and external light equipment, which might face the problems of cumbersome operation and low patient compliance during clinical translation. Based on the research trend, Gade et al. (2024) determined that the use of stainless steel MNs with a bevel angle of 60° could achieve the maximum scleral depth at an injection angle of 45°, maximizing the range of distribution of the drug within the sclera, and this study optimized the MNs on their own terms, expanding the range of action of the drug molecules. However, since the experiment was based on isolated sclera, the effects of variables such as blood flow and intraocular pressure in living tissue on drug distribution were not considered. In addition, if the stainless-steel material was replaced with a more biocompatible material, it might be possible to better balance the depth of MN probing with safety.

## 3 Microenvironmental molecule and signal detection

### 3.1 Glucose

Real-time monitoring and regulation of patient glucose levels had long posed significant challenges. The earliest application of MNs for insulin delivery was pioneered by McAllister in 2003, who successfully administered insulin through hollow MNs using external pressure (McAllister et al., 2003), resembling conventional injection methods. Subsequently, Prausnitz’s team experimentally demonstrated the superior pharmacokinetic properties of MNs in drug delivery (McAllister et al., 2003), establishing foundational theories for diabetes management systems.

Integrating insulin delivery with glucose monitoring necessitated the incorporation of micropumps and detection components on MN arrays. Ma first introduced piezoelectric (PZT) pumps for insulin transport (Ma et al., 2006), leveraging their advantages of ultra-low operating frequency, rapid response, and high reliability. Building on this, Meshkinfam’s team designed and simulated programmable insulin release processes (Meshkinfam and Rizvi, 2021). While these advancements propelled drug delivery technology, system integration complexities hindered manufacturing until the emergence of microfluidics-integrated MNs. This technology enabled precise fluid manipulation (flow rate, concentration, etc.) for rapid small-volume transport and mixing. Takeuchi’s integration of microfluidics with MNs achieved simultaneous glucose detection (Takeuchi et al., 2019), allowing real-time interstitial fluid analysis during drug delivery. Alternative approaches involving electrochemical conversion of glucose fluctuations into measurable currents have also shown promise.

**FIGURE 4:**
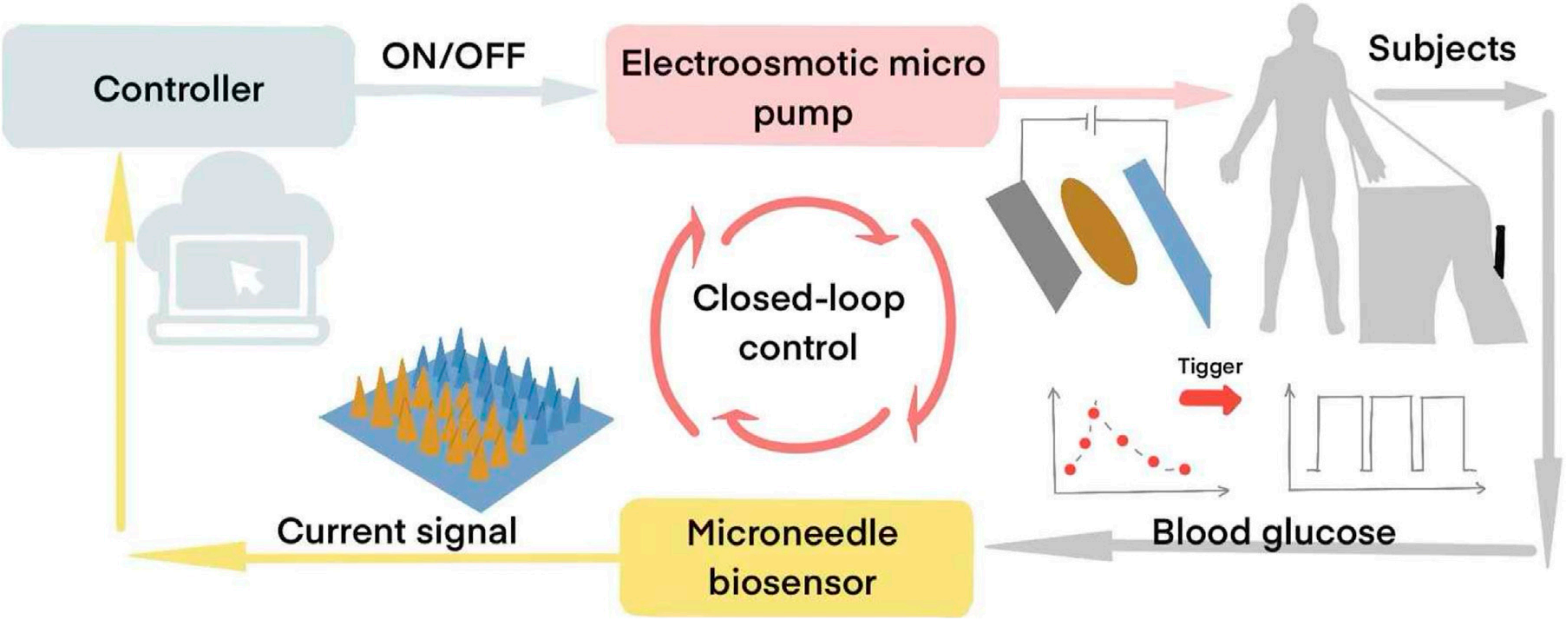
MN-based Closed-loop Glucose Monitoring and Regulation System (The graphene-PB modified MN electrode detects interstitial glucose levels and transmits signals to a PCB. When glucose exceeds a preset threshold, the PCB activates an electroosmotic micropump, releasing insulin from the reservoir via hollow MNs for 10 min. Glucose sensing then resumes, enabling alternating cycles of detection and insulin delivery until normoglycemia is restored) (Liu Y. et al., 2024).

Advancements in integration technologies led to Lee et al.'s development of graphene hybrid device arrays (Lee et al., 2016), incorporating sweat-control layers and temperature-responsive bioresorbable MNs for sweat-based glucose/pH monitoring and thermoresponsive drug release. Recently, Cui’s team (Liu Y. et al., 2024) engineered a wearable MN patch system featuring polystyrene hollow MN arrays penetrating the dermis, graphene biosensors, and chemically functionalized electroosmotic micropumps (Figure 4). This closed-loop system continuously monitored interstitial fluid glucose levels - which correlate strongly with plasma/serum biomarkers (Lee et al., 2016)– while delivering insulin through electroosmotic actuation, achieving glycemic control within 2 h without manual dosage calculation.

From simple injection improvements in the early 2000s to integrated monitoring modules and ultimately closed-loop systems combining glucose sensing with drug delivery, MN technology had evolved exponentially. Future enhancements in signal detection systems promised geometrically optimized patches capable of capturing molecular dynamics with heightened sensitivity, adaptable to diverse clinical scenarios. This progression heraldeda transformative era in autonomous diabetes management.

### 3.2 Cytochrome C

Cytochrome C (Cyt c) is one of the typical electron delivery carriers (Morse et al., 2024), which is crucial for understanding the metabolic state and apoptotic mechanism of microorganisms at the biofilm-material interface. The conventional methods for detecting cytochrome C content in the biomembrane interface microenvironment require a combination of proteomics, mass spectrometry, electron microscopy, etc. However, these methods are limited by the high cost of instruments and complex operating procedures, resulting in less-than-ideal detection efficiency for cytochrome C. Thanks to the excellent biocompatibility of MN materials and simple manufacturing process, An et al. (2025) used DNA/MeHA gels as the MN tip and optimized the MN fabrication parameters to ensure its mechanical strength and water absorption capacity, which allowed the MNs to penetrate the biomembrane. By combining fluorescence staining techniques, they effectively eliminated interference, enhancing the signal between cytochrome C and the MNs, significantly improving the sensitivity and accuracy of the detection. This study introduces a novel method for detecting cytochrome c (Cyt c) in the biofilm-material interfacial microenvironment with hydrogel MN arrays. The detection of interfacial microenvironments was critical to the understanding of complex physicochemical phenomena, but the detection of interfacial substances was a major challenge. Cyt c was critical to the understanding of microbial biofilm metabolism and apoptosis mechanisms. This new method for detecting cytochrome c (Cyt c) in the microenvironment of a biofilm - material interface using a hydrogel MN array enabled interfacial microenvironment detection, which was crucial for understanding complex physicochemical phenomena, but accurate and rapid detection of interfacial substances remained a major challenge.

### 3.3 Steroids

Cholesterol, as a major molecule in the composition of animal cell membranes, was of significance for the monitoring of cardiovascular and neurological diseases. Li Z. H. et al. (2023) added platinum (Pt) into the hollow MN material, which enabled the subsequent addition of cholesterol oxidase to couple to the surface of Pt. Finally, the cholesterol concentration was transmitted as an electrical signal through the platinum-enzyme sensor, resulting in reliable linear outcomes. The results showed that the MN sensor was capable of detecting cholesterol with high affinity and sensitivity over a dynamic range.

Cortisol, as an important component of the hypothalamic-pituitary-adrenal axis, cortisol dysregulation was often associated with stress disorders, anxiety, and Cushing’s syndrome. Measuring cortisol concentration in the interstitial skin fluid (ISF) might be useful to understand the changes in the human physiological state. Li et al. (Jing et al., 2024) used MNs as electrochemical biosensors to analyze the circadian rhythms of cortisol and to establish a correlation between cortisol in the interstitial skin fluid and blood cortisol levels.

### 3.4 Ketone bodies

Moonla et al. (2024) demonstrated the application of a wearable MN patch platform for continuous monitoring of β - hydroxybutyrate (BHB) levels in human interstitial skin fluid (ISF). The MN sensor was based on a gold-deposited platinum transducer modified with a polytoluidine blue O (poly - TBO) mediator layer, β-hydroxybutyrate dehydrogenase (HBD) and nicotinamide adenine dinucleotide (NAD^+^) cofactor. *In vitro* experiments showed that the sensor had high sensitivity, selectivity, stability, and good reproducibility for NADH and BHB detection. In human experiments, the MN sensor could dynamically track the changes of BHB levels in ISF after ketone beverage intake in healthy subjects, which was consistent with the trend of the results of the traditional blood test, but with a certain time lag. This MN sensor provideda promising means of dynamic BHB tracking for diabetic ketoacidosis management, personal nutrition, and health monitoring, and future in-depth studies of physiologic lag times and development of calibration algorithms were needed.

### 3.5 Processing of bioelectric signals

**FIGURE 5:**
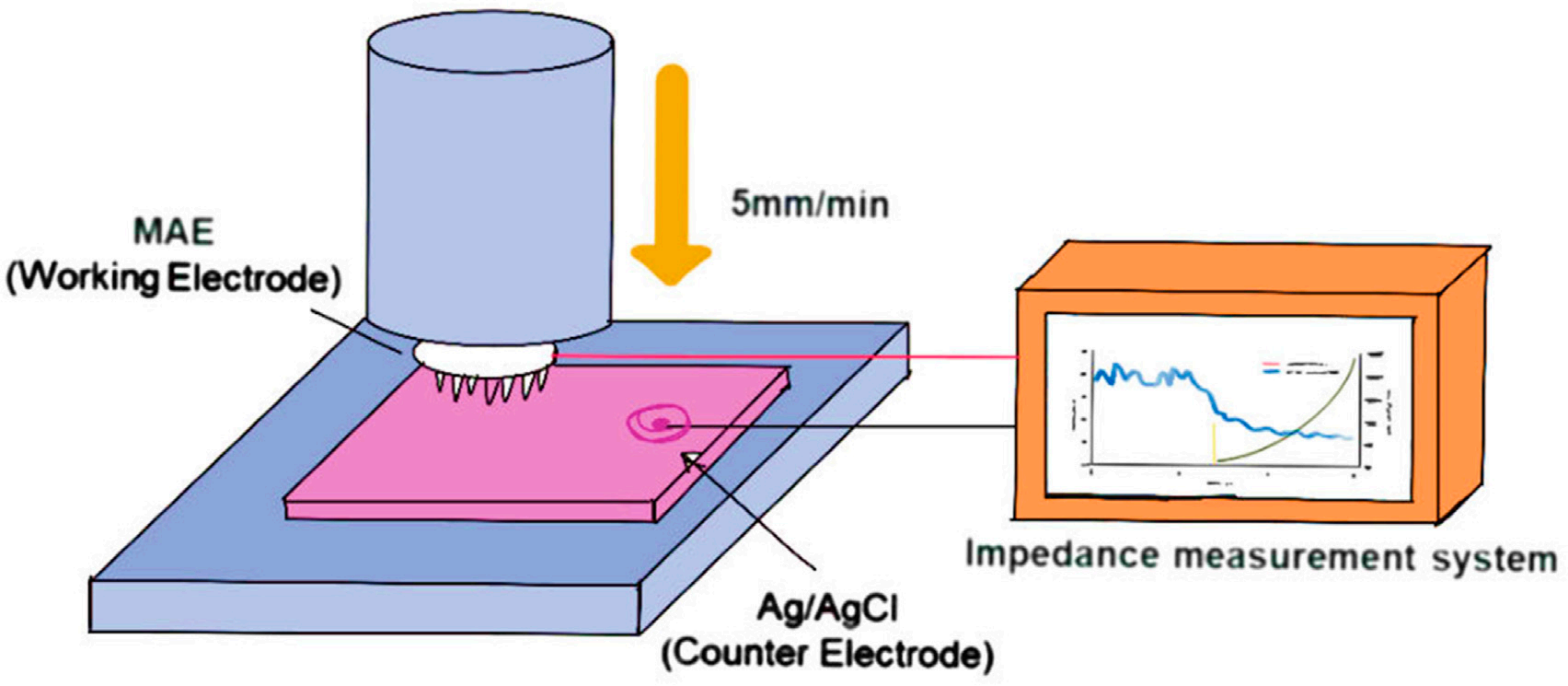
Schematic illustration of impedance measurement setup for MN array electrode (The fabricated Bi–In–Sn-based MN electrode (MAE) is vertically inserted into porcine skin tissue at a controlled speed of 5 mm/min. During insertion, the MAE is connected to an impedance measurement system to record real-time electrical impedance spectra, enabling evaluation of skin–electrode contact characteristics and electrical performance under dynamic loading conditions) (Gwak et al., 2023).

Human bioelectrical signals such as electrocardiogram (ECG), electromyogram (EMG) (Roberts et al., 2016) and electroencephalogram (EEG) (Opie et al., 2018) were important for the diagnosis and treatment of heart, brain and muscle-related disorders. Hyunjong et al. (Gwak et al., 2023) (Figure 5) used a Bi-In-Sn alloy-based ECG metal MN array to detect distinct P waves, QRS complexes, and T waves, meeting the requirements for electrocardiography. Building on this, (Liu Z. J. et al., 2024) developed multichannel MN dry electrode patches capable of distinguishing characteristic patterns of different diseases. The patch achieved single-MN-level accuracy with remarkable temporal and spatial resolution. Stable signals could be obviously obtained on the ECG recording tests of experimental rabbits, and multiple channels could show excellent synchronization.

When evaluating the mechanical contractile force and collecting electrophysiological signals of cardiac organoids, these organoids derived from induced pluripotent stem cells had gradually become an important model for assessing cardiac toxicity. However, real-time, *in situ* detection of the mechanical contractile force and electrophysiological signals of cardiac organoids remained a major challenge. Yin et al. (2025) proposed a sensory system based on micromotor arrays and resistive skin sensors. The cardiac-like organs were placed in the MEA chip while the sensors were controlled at the topmost part of the organs to achieve reliable and simultaneous electromechanical measurements, and electro-mechanical parameters were collected for evaluating the cardiac-like organs and tissues.

However, since MNs needed to accurately locate the sensor-tissue contact point, which would be very demanding on the operator’s skills, and miniaturized sensors might be interfered with by environmental noise, and multichannel systems needed to be supported by complex signal processing algorithms, data fusion was difficult, and the long-term electrochemical stability of the MN electrodes such as oxidation of alloys as well as the continued reliability of the sensors in organoid cultures still needed to be verified.

## 4 Tumor diagnosis and treatment

### 4.1 Melanoma diagnosis and treatment

**FIGURE 6:**
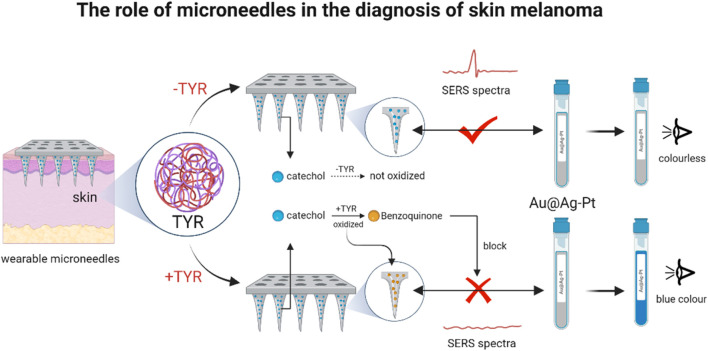
Dissolvable PEI MNs for delivery of STAT3 for siRNA-targeted treatment of melanoma (The MN arrays were functionalized with dopamine, enabling catechol-mediated binding of 4-MPBA-labeled Au@Ag-Pt nanozymes (M/Au@Ag-Pt) via stable borate esters to form MN/M/Au@Ag-Pt platforms. Upon insertion into TYR-containing skin, TYR catalyzed catechol oxidation to benzoquinone, disrupting nanozyme attachment and switching the SERS signal “off” and colorimetric signal “on.” Signal intensity showed a negative linear correlation with TYR levels, enabling dual-mode TYR quantification.) (reprinted with permission from Huang et al., 2023. Copyright 2023 American Chemical Society).

Characterized by easy recurrence, high mortality and high metastasis, cutaneous melanoma was one of the most aggressive skin cancers (van der Maaden et al., 2012). Tyrosinase (TYR) was a polyphenol oxidase essential for the synthesis of melanin, whose overexpression and accumulation in skin cells was considered to be an important marker of melanoma. Therefore TYR was an important biomarker for melanoma diagnosis (Zhou et al., 2016). However, how to capture the real changes of TYR in suspicious skin remained a major challenge. Based on this, for efficient detection of TYR (Figure 6), Huang et al. (2023) designed an Au@Ag-Pt nanoparticle wearable MN for screening of potential melanoma. The MN was designed based on the principle that in the presence of TYR, catechol immobilized on MNs are preferentially oxidized to benzoquinone, which competitively hinders the interaction between MN and Au@Ag-Pt NPs, thereby triggering the SERS-colorimetric signal inter-switching. More importantly, the MN patch had demonstrated flexible and stretchable properties and could be firmly adhered to the skin without causing chemical or physical irritation. It was further shown that it has important clinical significance in the early diagnosis and monitoring of cutaneous melanoma.

Based on the MN, an ultrafine microplatform, Pan et al. (2018) fabricated biologically safe dissolving polyethyleneimine (PEI) MNs for delivery of siRNA for STAT3 for targeted treatment of melanoma. STAT3 was a signal transducer and transcriptional activator associated with malignant cellular behaviors. SiRNAs were small interfering RNAs that cause gene silencing of the target gene and inhibit gene expression. This MN inserted into the skin could locally form a STAT3 siRNA-PEI complex to mediate the silencing of the STAT3 gene and directly inhibit the proliferation of melanoma, which had been shown to have good efficacy in mice.

### 4.2 Diagnosis of breast cancer

Breast cancer is one of the most common tumors threatening women’s health worldwide. In the diagnosis of breast cancer, the key is to detect epidermalgrowthfactorreceptor2 (ErbB2), a biomarker of breast cancer. Therefore, Dervisevic et al. (2021) designed a high-density gold-plated silicon MN array for ErbB2, which functioned as both a biomarker extractor and an electrochemical sensor, and was capable of selectively capturing ErbB2 with linearity over a concentration range of 10–250 μg-L^-1^, resulting in effective detection of ErbB2. The study opened up a new direction for the development of high-performance wearable nursing devices.

## 5 Treatment of skin-related diseases

### 5.1 Wound healing

To accelerate wound healing in patients, researchers had designed a two-phase MN array that worked by mechanically interlocking the expandable MN tip with the skin tissue, which achieved about a 3.5-fold increase in adhesion strength in skin wound healing compared to traditional chemical adhesives. The MN array was more convenient and less time-consuming than suturing wounds, prevented gas or liquid leakage, and reduced tissue damage by evenly distributing the applied mechanical stress. It was worth noting that the MNs could also be combined with transdermal drug delivery to accelerate wound healing by delivering substances such as anti-inflammatory and crude regenerative molecules to the target site through reversible microchannels. The experimental results showed that the MN array could improve wound healing in diabetic patients by increasing the delivery efficiency. At the same time, Liu et al. (2025) demonstrated that MNs could respond to reactive oxygen species (ROS) in the diabetic microenvironment and subsequently produced oxygen (O_2_) and nitric oxide (NO). These gases comprehensively promoted neurovascular regeneration, reduced oxidative stress levels, and reduced inflammation.

In addition, the material of the MN itself might be a good solution to the wound problem. As a two-dimensional inorganic chemosynthetic material, MXenethe could damage the bacterial membrane in direct contact with the bacteria (Seidi et al., 2023), achieving the therapeutic efficacy of antibacterial promotion of wound healing. When MXene as a MN base material, together with the other antimicrobial drug filling, this kind of MN patches for wound healing provided a new strategy (Hu et al., 2024).

### 5.2 Scar repair

**FIGURE 7:**
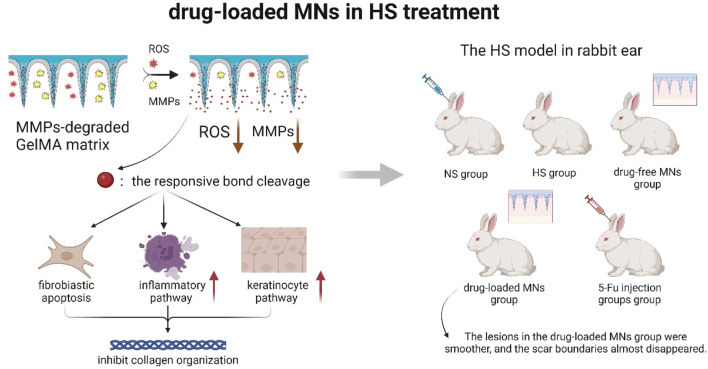
Schematic diagram of drug-loaded MNs remodeling the pathological microenvironment of scar tissue (A ROS- and MMPs-responsive separating MNs was developed by UV-crosslinking GelMA with a 5-FuA prodrug for sustained *in situ* drug release. The MNs tips respond to elevated ROS and MMP-2/9 in HS, triggering localized 5-FuA release that promotes fibroblast apoptosis, modulates inflammatory and keratinocyte pathways, and inhibits collagen over-deposition. Using a rabbit ear HS model verified that the relationship between MNs drug loading and scar thickness) (Yang et al., 2024).

Yang et al. (2024) (Figure 7) made the hypothesis that drug-carrying MNs could inhibit fibroblast regeneration and mediate keratinocyte differentiation to reduce collagen fiber production. They experimentally demonstrated that drug-carrying MNs could remodel the pathological microenvironment of the scar tissue through reactive oxygen species (ROS) scavenging and depletion of matrix metalloproteinases (MMP). It revealed the potential mechanism of repairing scar and provided new ideas to clinically solve this shape problem of patients. However, the study lacked long-term follow-up data beyond 6 months, the effect of the degradation products of the MN material on the tissue had not been clarified, and the technique did not involve studies of individualized dosing regimens for patients with different skin types and scarring stages, so clinical applicability was not yet certain.

### 5.3 Pigmentation

Sawutdeechaikul et al. (2021) conducted a study on dissolvable and detachable MNs loaded with glutathione and vitamin C. To exclude factors such as infection, inflammation, and immune cell regulation, they selected volunteers some time after acne occurrence. The volunteers used the MNs daily, and their melanin index was measured using a skin colorimeter. After 4 weeks, the MN treatment group showed a significant reduction in melanin compared to the control group. The authors did not explore the feasibility of simplifying the frequency of administration, although they concluded that daily microneedling might affect patient tolerance, and the subject population was limited to the post-acne hyperpigmentation group, with no verification of generalizability to other types of pigmentation such as melasma and photodamaged spots.

### 5.4 Skin aging

Skin aging is characterized by endogenous factors of heredity and genes, and also accepted the influence of exogenous factors such as environmental exposure, nutritional intake, etc. These combined factors ultimately led to the disruption of the cellular microenvironment, and the decline in the water content of the extracellular matrix, causing the slowing down of the cellular metabolism and the reduction of elasticity and collagen fibers, which accelerated the aging process of the skin (Li S. Z. et al., 2023). Researchers used polysaccharides and proteins to create a three-dimensional network structure, and hydrogel MNs made of this material could well provide a growth environment for the cells again, improved the adhesion, proliferation, and differentiation of the senescent cells, and slowed down the aging process (Zhang et al., 2025).

### 5.5 Treatment of psoriasis

Zhou et al. (2023) used iontophoresis-driven MN patches loaded with dexamethasone sodium phosphate (Dex) for the treatment of psoriasis in mice and found that typical signs of psoriasis, such as dryness, thickening, erythema, and scaly skin, disappeared after 5 days, where dexamethasone sodium phosphate was able to be released consistently and stably. Le Thanh et al. (2014) designed heparin-coated porous MNs (HPMN) to capture and remove the inflammatory chemokine MCP-1 and deplete inflammatory monocytes to alleviate persistent inflammation. Heparin was covalently cross-linked to the surface of the MNs to enhance chemokine capture, while the porous structure of the MNs served as channels for monocyte infiltration. In a psoriasis mouse model, the treatment resulted in a reduction in epidermal thickness and immune cell infiltration. However, while Zhou was biased towards the optimization of drug delivery, Le was biased towards optimizing the structure of MNs. In the future, electrically responsive smart MNs could be constructed in psoriasis treatment, which was expected to achieve on-demand drug release that could be triggered according to pH or inflammatory factor concentration, while targeting to regulate the immune microenvironment to enhance the precision and safety of psoriasis treatment.

### 5.6 Resolution of androgenetic alopecia

For androgenetic alopecia (AGA), PRP - MNs currently provided painless, minimally invasive and sustainable PRP-promoted hair growth (Sun et al., 2024); AR - PROTAC - MNs showed highly biocompatible, one-step delivery and long-lasting efficacy without systemic toxicity or androgen deficiency-related disorders (Wang et al., 2023); DMN - VPA maximized VPA delivery while promoting hair follicle regeneration; there were also multiple MNs that exhibited different roles and limitations in AGA treatment (Fakhraei Lahiji et al., 2018). Additionally, some studies had explored the use of MNs for delivering drugs or other bioactive compounds to promote hair regeneration. For example, MNs-loaded drug delivery systems had been further developed for the treatment of AGA. Other research have investigated the use of conditioned medium (CM) (Yuan et al., 2022), stem cell-derived exosomes (Yang et al., 2019), and other bioactive compounds for therapeutic purposes.

## 6 Others

### 6.1 Treatment of neurodegenerative diseases

**FIGURE 8:**
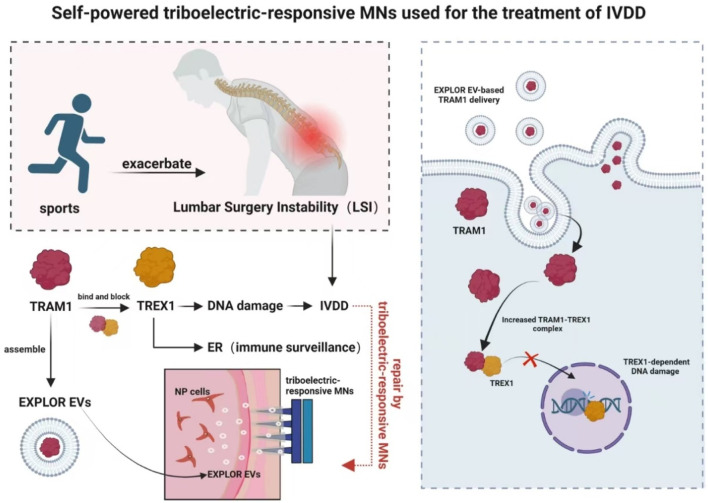
Schematic diagram of a self-powered triboelectric-responsive MNs system for the treatment of intervertebral disc degeneration (The MNs system integrates a triboelectric nanogenerator (TENG) composed of polytetrafluoroethylene (PTFE) and indium tin oxide (ITO) friction layers, coupled with polypyrrole (PPy)-coated MNs to convert mechanical energy into electrical stimulation. During exercise, the system generates triboelectric signals that trigger the on-demand release of optogenetically engineered extracellular vesicles (EXPLOR-EVs) loaded with TRAM1 protein. These EVs function to restore endoplasmic reticulum (ER) localization of TREX1, inhibit nuclear DNA damage, suppress cGAS-STING pathway activation, and thereby mitigate inflammation-associated IVDD progression) (Zhang W. F. et al., 2024).

Zhang W. F. et al. (2024) (Figure 8) fabricated a self-powered triboelectric-responsive MNs system that could be used for sustained release of engineered extracellular vesicles as an alternative to traditional NSAIDs and traditional surgery for disc degeneration. They searched for inhibitory validation of new targets and mechanisms, which had clinical potentials for the treatment of degenerative diseases. Although this study improved TRAM1 protein loading efficiency using the EXPLOR technology, payload loading and vesicle engineering for extracellular vesicles in biological targeted therapy might still face challenges that required further optimization to enhanced therapeutic outcomes. Despite the promising preclinical results of tissue engineering strategies, the lack of integration between etiology-driven interventions and endogenous therapeutic mechanisms targeting degenerative molecules had prevented any method from being approved for clinical application to date.

### 6.2 Exploration of the functionality of the MN chip system

Early liver chips ignored the three-vessel structure and even the dynamic flow that had been shown to promote cell function and long-term culture. Liver chips with single-flow pathways as a vascular alternative later emerged to provide oxygen and nutrients to cultured cells and remove waste products. However, single-vessel structures were difficult to create physiologically similar oxygen and nutrient gradients in the cell culture zone, which was considered one of the main factors leading to the differentiation of functional zones of the liver alveoli. To deal with this deficiency, Li S. B. et al. (2023) constructed MN arrays that led to the formation of a perfusable hepatic sinusoids, which facilitated the flow of fluids in the culture zone and hepatic metabolism. The pioneering experiment provided new ideas for organoid development.

## 7 Future direction and outlook

MNs, as an innovative transdermal delivery system, have demonstrated remarkable progress in recent years across drug delivery, vaccination, disease diagnosis, and chronic disease management. By penetrating the stratum corneum with micron-sized needles, they enable painless and minimally invasive delivery of drugs or biomolecules while avoiding first-pass metabolism and systemic side effects. Current applications include successful delivery of insulin, vaccines (e.g., influenza and rabies vaccines), antifungal agents, and local anesthetics, with emerging potential in diabetes glucose monitoring and cancer immunotherapy. Clinical trials confirm that MNs enhance patient compliance, particularly benefiting pediatric and geriatric populations, as well as those with needle phobia.

However, technological advancement inevitably faces challenges. Balancing material selection and structural design remains critical. MNs must maintain mechanical strength (for stratum corneum penetration) while ensuring biocompatibility (to prevent irritation or premature degradation). Existing materials—including silicon, metals, and hydrogels—present limitations in strength, degradation rate, or drug-loading capacity. For instance, hydrogel-based MNs offer dissolvable properties but constrained drug-loading capacity, whereas silicon MNs exhibit superior strength yet require post-application removal due to non-degradability. Developing composite materials with high drug-loading efficiency, tunable degradation profiles, and optimal skin compatibility could address these constraints. Furthermore, achieving spatiotemporal control over drug release kinetics and penetration depth remains technically challenging. In vaccine delivery, for example, rapid antigen release may compromise immunogenicity, while delayed release risks local inflammation. Emerging solutions involve stimuli-responsive MN designs (e.g., pH, temperature, or enzyme-activated materials) to achieve on-demand biomolecule release.

Manufacturing scalability and quality control present another frontier. While traditional fabrication methods like photolithography and molding face high costs and suboptimal yield, next-generation techniques such as 3D printing and soft lithography show promise for high-throughput, cost-effective production. Standardized quality assessment protocols—evaluating mechanical integrity, drug-loading uniformity, and stability during storage/transportation—are urgently needed to ensure clinical viability. Cost optimization in manufacturing will further expand MN applications.

Capitalizing on their adaptability and chip-integration potential, future MNs are poised to revolutionize precision medicine through multifunctional integration. Converging with microfluidic chips and biosensors could enable closed-loop “sample-to-therapy” systems that analyze biomarkers (e.g., glucose, inflammatory cytokines) and deliver tailored therapeutics. Synergy with gene/cell delivery technologies may enhance MN-mediated transport of CRISPR-Cas9, mRNA vaccines, or stem cells, propelling advances in gene therapy and regenerative medicine. As biocompatible materials evolve, these versatile platforms are anticipated to emerge as safe, efficient solutions across diverse medical scenarios.
